# Minimizing Stress Shielding and Cement Damage in Cemented Femoral Component of a Hip Prosthesis through Computational Design Optimization

**DOI:** 10.1155/2017/8437956

**Published:** 2017-02-28

**Authors:** Abdellah Ait Moussa, Justin Fischer, Rohan Yadav, Morshed Khandaker

**Affiliations:** Department of Engineering and Physics, University of Central Oklahoma, Edmond, OK, USA

## Abstract

The average life expectancy of many people undergoing total hip replacement (THR) exceeds twenty-five years and the demand for implants that increase the load-bearing capability of the bone without affecting the short- or long-term stability of the prosthesis is high. Mechanical failure owing to cement damage and stress shielding of the bone are the main factors affecting the long-term survival of cemented hip prostheses and implant design must realistically adjust to balance between these two conflicting effects. In the following analysis we introduce a novel methodology to achieve this objective, the numerical technique combines automatic and realistic modeling of the implant and embedding medium, and finite element analysis to assess the levels of stress shielding and cement damage and, finally, global optimization, using orthogonal arrays and probabilistic restarts, were used. Applications to implants, fabricated using a homogeneous material and a functionally graded material, were presented.

## 1. Introduction

Between the two alternatives of total hip replacements, the cemented fixation method was mostly adopted owing to offering the immediate stability from cement-stem and cement-bone bonding interfaces after implant surgery [1, 2]. Clinical studies however reported that cemented hip prostheses fail to function properly due to the loosening of the fixations after long-term use [3, 4]; debonding of the cement-stem and cement-bone interfaces and the local fractures in the cement mantle were perceived as the primary mechanisms of cemented hip implant loosening [4]. Improved cementing techniques had then been developed to reduce the prevalence of loosening and to guarantee long-term fixation of the prosthesis [5–9]. Stress shielding of the bone received increased attention over the years [10]; whether cemented or uncemented implants were used, mechanical loosening owing to the physiological dynamic response of the bone is one of the main factors affecting the implants long-term durability. The presence of the femoral implant in the intramedullary canal reduces the loads normally applied to the bone of the proximal femur, resulting in periprosthetic bone resorption, bone loss, cortical bone thinning, and joint prosthesis failure [11–15]. Revision surgery to address such failure involves increased risks, complications, and costs. Given that the average life expectancy of many patients undergoing total hip arthroplasty (THA) exceeds 25 years, ongoing stress shielding is clinically important and the demand for implants which maximize the load-bearing capabilities of the bone is high [16, 17].

Implant design and material properties have great effects on the total hip joint stability and long-term performance. For instance, if the stem design and material lead to high stresses in the fixation area of the prosthesis, local fractures or fatigue failure of the cement is quite likely to occur. Observations from fatigue experiments and clinical studies had attributed the loosening of the cement-stem fixation to the local fractures initiated in the cement mantle adjacent to the stem that gradually propagated and produced separation of the stem-cement interfaces [4, 18, 19]. Concurrent with cement damage and stress shielding other studies revealed that stiffer implants induce high levels of stress shielding over the proximal femur and low levels of interface stress among the femur-implant constituents [20, 21], while in contrast less stress shielding and high levels of cement damage were observed with low stiffness implants [22].

The long-term survival of the cemented prosthesis is contingent on achieving a balance between stress shielding and cement damage; to this end a number of studies investigated several prospective modifications. In some of these investigations less stress shielding and improved implant stability were observed in fully cemented fixations [23] and with poly-methyl methacrylate (PMMA) instead of other bioactive bone cements [24]. It was also revealed that shorter stems had improved the overall stability of the cemented fixation and provided the femur with more proximal load [25]. Implant shape modification techniques were also investigated in cemented and cementless fixations; common design optimizations in the former fixation targeted the cement layer or cement-prosthesis interface with the objective of minimizing stress concentration in these areas [26–28]; however stress shielding of the bone was not quantified for use in the design analysis. Several shape optimization models were developed for cementless prostheses where one [27, 29–32] or more [33–35] performance criteria were used in the search method. In the most relevant of these studies [35], a three-dimensional model of the implant based on the commercial Tri-Lock (Depuy, Inc., Warsaw, IN, USA) was constructed using suitable interpolation between a fixed number of key cross sections and a simulation based structural optimization was used to identify new and improved designs. The major drawbacks of this form of optimization are the computing cost which is usually expensive; additionally they are prone to a risk of being trapped in local optima and the CAD interpretation of the shape optimization result is not trivial [36].

In the current study, we introduce a novel evolutionary technique to optimizing stem designs in a cemented hip prosthesis with the objective of minimizing stress shielding and cement damage. The self-regulated technique combines realistic and solid modeling of the implant and embedding medium, finite element to assess the levels of stress shielding, and cement damage in addition to a fast global optimization using orthogonal arrays and probabilistic restarts.

This paper is structured as follows; in Section 2 we introduce the computational technique used to realistically optimize the geometry of the stem of a cemented hip prosthesis. In Section 3, we apply the methodology thus introduced to identify the optimal stem design given two types of materials, Titanium alloy and a longitudinally inhomogeneous functionally graded material. In the final section, we conclude with a summary and discussion of future work.

## 2. The Computational Technique

In this section, we shall describe the general structure of the computational technique used to model and optimize the shape of a cemented hip implant with the objective of minimizing bone stress shielding and cement damage.

### 2.1. The Modeling of the Prosthesis

**Figure 1:**
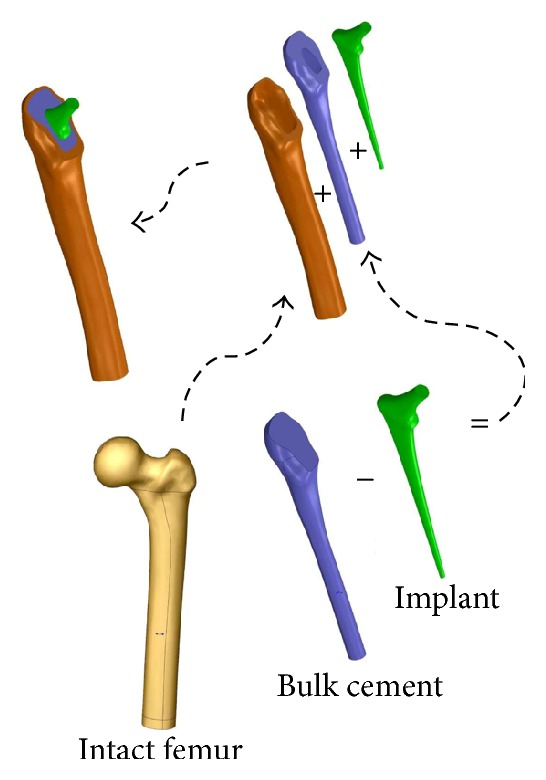
The intact femur bone was cut at its proximal part to allow for insertion of the implant. The geometry of the implant was deducted from the bulk cement represented and fixed length of the void inside the cortical bone. The implant and updated cement geometry were then aligned with the axis of the cortical bone and a femur-cement-implant construct was formed.

Our femur-prosthesis model is depicted in Figure 1, it consisted of a cortical bone representing the femur, the bone was cut at its proximal part to allow for insertion of the implant, and the cancellous bone was excluded for simplification. The implant was centered with the bone at its distal end and aligned with the axis of the femur; the cement filled the space between the implant and the bone and extended over the length of the stem. The cortical bone model was acquired from Sawbone Inc.

**Figure 2:**
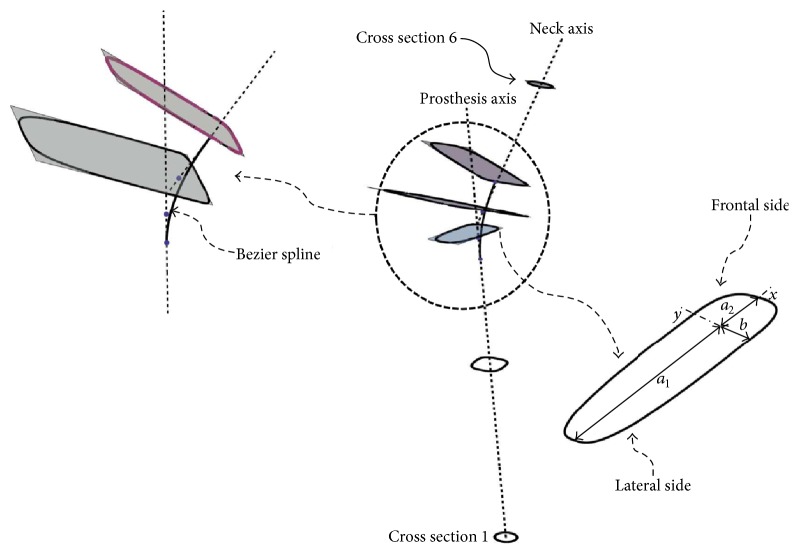
Skeleton of the hip implant. Each cross section is characterized by three geometrical parameters (*a*_1_, *a*_2_, *b*) in addition to the exponent (*p*) in (1). A four-point Bezier spline interpolated the sharp transition between the neck and prosthesis axes.

To accurately reproduce the geometry of the implant, we used a combination of mathematical and solid modeling. We flushed the sharp transition from the neck to the prosthesis axis with a four-point Bezier spline as depicted in Figure 2; we then selected 6 key cross sections with the centers and normal unit vectors, respectively, tangential to the prosthesis axis (cross sections 1, 2, and 3), the Bezier spline (cross section 4), and the neck axis (cross sections 5 and 6) as depicted on the same figure. The cross section on the distal end was circular with a fixed radius, the rest of the cross sections had variable shapes and sizes, and the characteristics equations of the cross-sectional profiles were modeled using (1) [35] where (*a*, *b*) are the lengths of the semiminor and semimajor axes in a local system of coordinates described by the *x*- and *y*-axes as depicted on the scaled up view of one of the cross sections; the exponent (*p*) is an integer number.

(1) $$\begin{array}{c} {\left(\;\frac{x}{a}\;\right)}^{p}+{\left(\;\frac{y}{b}\;\right)}^{p}=1. \end{array}$$

**Figure 3:**
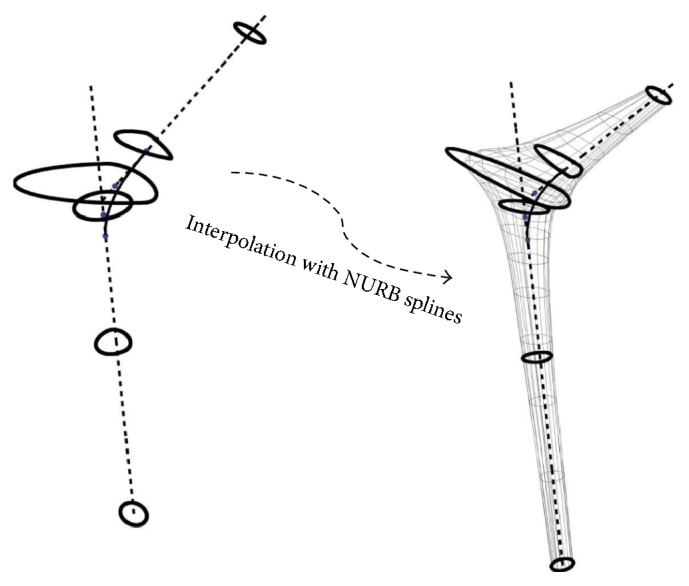
A series of NURB splines interpolated between the skeleton cross sections (left) to produce the hip model shown on the right.

For more control over the shape and size of a cross section, the frontal and lateral sides were modeled according to (1), the sides shared the same exponent (*p*) and had identical lengths of the common semiaxis, and they may however have different lengths assigned to the opposite semiaxes as depicted in Figure 2. A total of four design variables (*a*_1_, *a*_2_, *b*, *p*) characterized the shape of each of the five cross sections, and together twenty parameters characterized the skeleton of the stem. A series of third-order Nonuniform Rational Basis Splines (NURBS) was interpolated between the different cross sections to produce the mathematical model depicted in Figure 3. The mathematical modeling of the stem was completed in Mathematica and different geometries are produced upon changes in the parameters of the cross sections.

**Figure 4:**
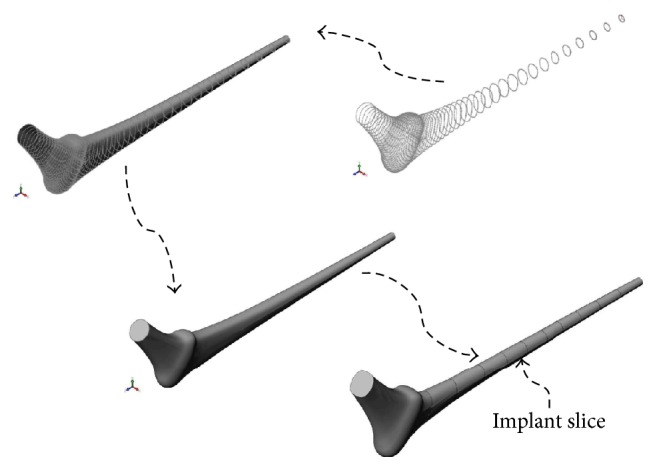
A fixed number of points were imported to the graphical interface of SolidWorks and interpolated with splines (top right), a closed surface envelope was mapped over the splines including the caps on both ends (top left), the enclosure was then filled, and a solid model of the implant was formed. The lengthwise slicing on the bottom right is optional.

To produce the accompanying solid model, we developed a program that imported the coordinates of a fixed but sufficient number of points on the surface of the implant model thus constructed to the graphical interface of SolidWorks; the flexibility of the structural programming in this software allowed for automatic interpolation among selected points along with surface mapping, solid modeling, and the optional lengthwise slicing of the implant as described in Figure 4. Using the same software, we successfully subtracted the material of the implant from the bulk cement and aligned the femur model together with the updated cement and implant geometries to produce the cemented femur-prosthesis model depicted in Figure 1. The procedure was automated to work with different prostheses models upon changes in the geometrical design parameters.

### 2.2. The Finite Element Analysis

**Figure 5:**
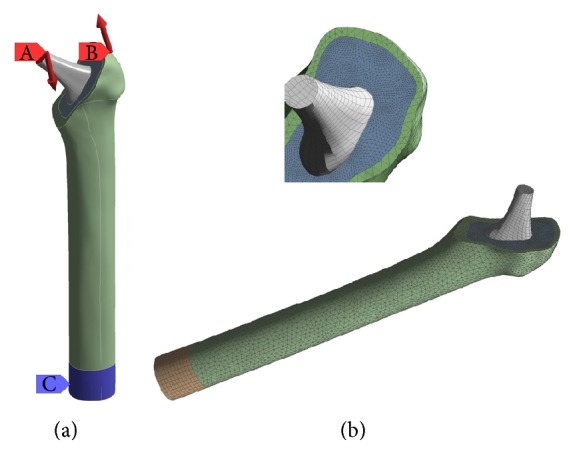
(a): (A) body force, (B) abductor muscle force, and (C) fixed support. (b): unstructured mesh with a combination of Tetrahedron, Patch Conforming, and Sweep Method was used for the assembly.

Previous studies [37] on the ability of computational methods to predict fatigue cracking in experimental models of a simplified femur structure showed that continuum damage mechanics can reliably predict cement fatigue locations in a timescale comparable with experimentation. Further studies [38] showed that a loading configuration including the hip joint contact force and the abductor force can adequately reproduce in vivo loading of cemented total reconstructions. Hereupon, a stress life fatigue analysis with constant amplitude (body load) and proportional loading, simulating average human walking condition, was conducted on the hip assembly thus constructed. The simulation setup in Figure 5(a) is similar to that of Jeffers et al. [37]; it allowed a joint force of 2.5 kN to be applied at 10° angle from the axis of the femur and an abductor force of 1.5 kN at an angle of 15° together equivalent to 3.5 times bodyweight loading (assuming bodyweight of 700 N). Bonded and rough (no sliding) fixations were used at the bone/cement and cement/implant interfaces, respectively.

An unstructured mesh with a combination of Tetrahedron, Patch Conforming, and Sweep Method was used for the volumes of the implant, cement, and bone, the mesh was refined around the implant and on the boundary between the cement and bone, and a sample representation of this mesh is shown in Figure 5(b). We maintained the same global and local mesh controls in ANSYS Workbench 16.0 to ensure consistency of mesh element size among all models upon changes in geometry and/or material stiffness. The bone and PMMA cement were assumed homogeneous, isotropic, and linear elastic with modulus of elasticity of 18.6 GPa and 2.28 GPa, respectively; the Poisson's ratio was assumed constant among all materials and equal to 0.3 [39].

To estimate stress shielding of the bone, we used (2), where (*σ*_*i*_, *σ*_*i*_′) are the calculated equivalent alternating (Von Mises) stresses, respectively, on a point of index (*i*) on the external surface of the intact femur in Figure 1 and a femur with implant. The index (*i*) runs from (*i* = 1) to (*i* = *N* = 3040) preselected locations along the length and around the femur model. *σ*_av_ is the average equivalent alternating stress over the external surface of the intact femur; the smaller the index, the less effective the stress shielding of the implanted bone.

(2) $$\begin{array}{c} \text{Stress Shielding Coefficient}=\frac{1}{\sigma_{av}}\left[\;\overset{N}{\underset{i=1}{\sum }}{\left(\;\sigma_{i}-\sigma_{i}^{\prime }\;\right)}^{2}\;\right]. \end{array}$$

The total damage accumulation in the cement was evaluated by summing the local damage over the points in the cement nodes following (3), where (*d*_*i*_) is the local fatigue damage at node (*i*) and (*i* max) is the total number of cement nodes. In calculating (*d*_*i*_), we first used the Goodman criterion to correct for mean stress effects; the Von Mises stress was then used to convert the multiaxial stress to a single valued stress and the *S*-*N* curve of Davies et al. [40] was used to estimate the number of cycles to life (*N*_*i*_) at mesh node (*i*). *d*_*i*_ was then assigned a value of 1 if the number of cycles to life is less than the design life and 0 otherwise. The term critical cement damage accumulation parameter was used as the failure index representing the percentage of the cement damaged at the design life of 2 million cycles [41–43].

(3) Critical  Cement  Damage  Accumulation  Parameter=∑i=1i maxdi.

**Figure 6:**
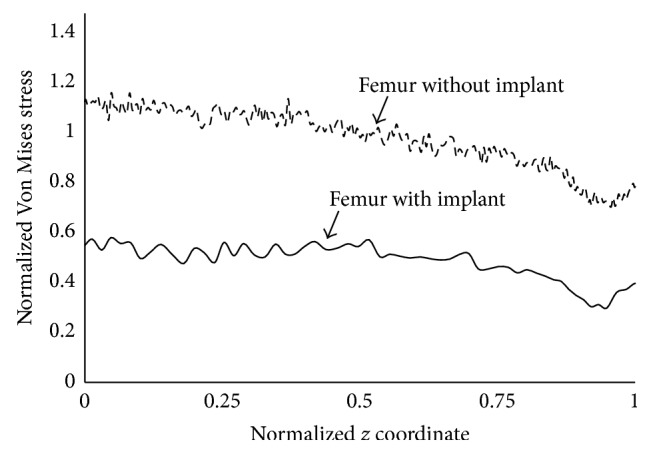
Stress distribution (moving average) over the surface of the cortical bone.

**Figure 7:**
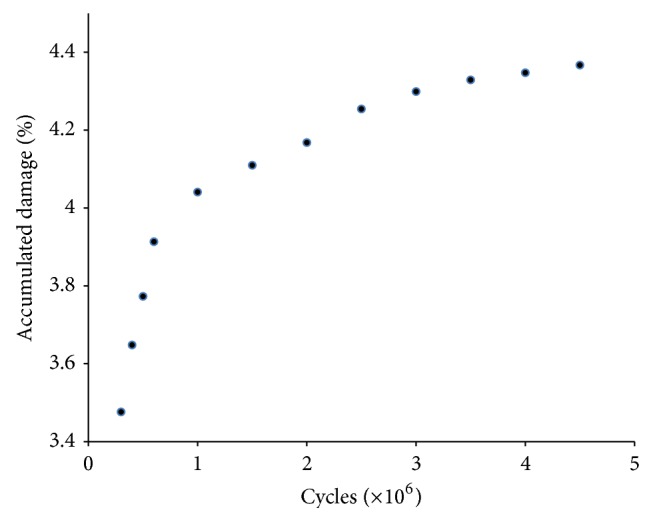
Accumulated damage within the cement as a function of the number of loading cycles (design life of 10^9^ cycles). The growth rate of fatigue damage is rapid and significant at early loading cycles gradually decreasing in subsequent stages of the loading.

Stress distribution over the external surface of the bone for the intact femur in Figure 1 and a femur with Titanium implant (Titanium alloy Ti-6Al-4V with modulus of elasticity of 110 GPa and a Poisson's ratio of 0.3 [39]) were plotted in Figure 6, the abscissa refers to the length from the distal end of the implant, the ordinate pertains to the normalized stress at that location, and the normalization constant was taken as the average stress on the intact femur. Stress shielding of the bone with implant is clearly noticeable owing to the difference in stress distribution averaging between 40% and 50%; these percentages are consistent with experimental measurements [44]. The accumulated cement damage was plotted in Figure 7 as a function of the number of cycles. The growth rate of the fatigue damage is rapid and significant at early loading cycles as expected, gradually decreasing in subsequent stages of the loading. Inspection also indicated that the most likely sites for failure initiation were in the proximal anterior region and at the distal tip of the prosthesis; such fatigue scenario is generally consistent with experimentation [41–43].

The setup of the finite element analysis (FEA), including the application of the body and abductor force, interface boundary conditions, material properties, assembly meshing, the setup, and solution of the fatigue analysis, was automated using a self-developed script for the ANSYS Workbench-Mechanical; the script is called every time the geometry and/or the material stiffness distribution were updated. Following geometrical updates of the implant, the ANSYS software exports data files with the calculated Von Mises stress over the bone surface and the calculated damage in the cement nodal points; these results are used to estimate the stress shielding coefficient and the critical cement damage accumulation parameter used in the optimization described in Section 2.3.

### 2.3. Optimization Setup and Procedure

Up to this point of the study, we successfully completed the mathematical modeling and programing of a model hip implant, the automatic reconstruction of a solid model that matches the geometry of the prosthesis; last but not least, we performed a fatigue analysis under average human walking condition in a self-regulated manner and were able to assess stress shielding through the stress shielding coefficient in (2) and the cement damage from the critical cement damage accumulation parameter in (3). Each element in this scenario can be rerun and different results may be achieved depending on the stem geometry and material properties. We initiated a self-regulated optimization process that reconfigured the geometry of the implant in a cemented prosthesis with the objective of minimizing a cost function defined as the sum of the squares of the normalized stress shielding coefficient and critical cement damage accumulation parameter; the normalization constants in (4) were, respectively, the maximum stress shielding coefficient and maximum critical cement damage accumulation parameter among all the design of experiments used throughout the optimization. The rationale of this technique is explained next.

(4) Objective  function=accu  damage  ratemax  accu  damage  rate2+stress  shielding  coefmax  stress  shielding  coef2.

We identified twenty geometrical parameters among five cross sections (2 through 6 in Figure 2) that together control the geometry of the stem and cement mantle; fifteen of these parameters including the size of the principal axes in each cross section (*a*_1_, *a*_2_, and *b* in Figure 2) were set to take on any real value within their respective search intervals; the exponent (*p*) in (1) on the other hand was set to take on integer values alone. The design boundaries of the semimajor and semiminor axes were set so the cement thickness around the implant is maintained between 2.0 and 3.0 mm; additional constraints were added so the produced stem designs were clinically admissible.

**Figure 8:**
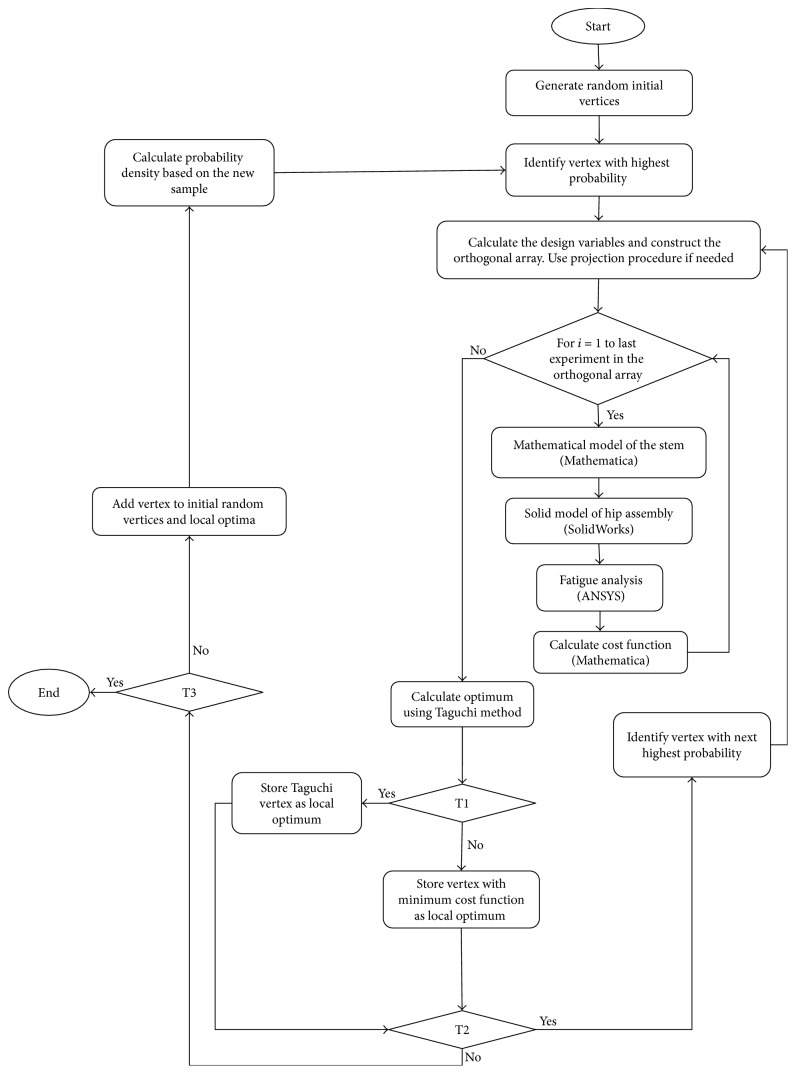
Global optimization. T1: Taguchi suggested optimum is best; T2: already known as an optimum; T3: maximum number of analyses is reached.

Given the multiplicity of the design variables a fractional factorial based-design optimization following the Taguchi algorithm [45] was used; the methodology schematics is diagrammatically explained in Figure 8. We began with a fixed number of random vertices, where each vertex represented a possible geometrical configuration of the implant equivalent to twenty random values within the constrained boundaries of the design variables. We identified the vertex that is the farthest from the rest of the vertices using the variable variance probability (VVP) density defined in the appendix. For each parameter (*x*_*i*_) in the vertex, we introduced two design values (*x*_*i*_^1^, *x*_*i*_^2^); we used (5) for the nonintegers, where Δ*x*_*i*_ refers to the size of the domain of analysis of variable (*x*_*i*_), and (6) was used for the integer exponents (*p*_*i*_). With the design variables thus introduced, we initiated a two-level orthogonal array optimization following the Taguchi algorithm [45] to identify the combination of design variable (optimum vertex) that locally minimize the cost function in (4). The optimum vertex thus found was then added to the initial random vertices so to avoid selecting the same experiments and the process was repeated for the next optimum; the VVP ensures the globalization of the search since the next vertex selection is based on the largest distance among all vertices. There may be cases, however, when the new optimum is identical to one of the stored optima, that the suggested optimum is not better than one of the current vertices because of factor interactions, or that one or more of the vertex levels are not within the design space. In cases like these, we proceed as indicated in the diagram of Figure 8. The box projection procedure in (7) ensures that the levels are always selected within their search intervals.

(5) xik=xi∓0.2Δxik=1,2,  i=1  to  2

(6) pik=pi∓1k=1,2,  i=1  to  20

(7) xi=xilower  boundif  x<xilower  boundxiupper  boundif  x>xiupper  bound.

## 3. Applications

In this section we shall represent the results of the application of the technique introduced in Section 2 to a cemented Titanium alloy and a functionally graded implant.

### 3.1. Optimization Setup

**Figure 9:**
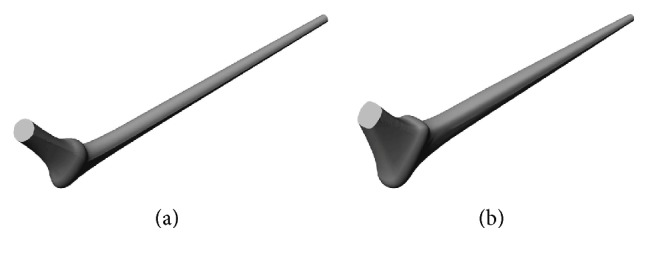
Lowest (a) and uppermost (b) implant configurations.

The radius of the circular cross section at the distal end was set to a fixed value of 6 mm, the total length of the implant was *L* = 185 mm, the upper and lower bounds of the exponent in (1) were set according to the first two rows in Table 1 for the cross sections in Figure 2, and a value of 2 corresponds to a circular or oval cross section, while values of 3 and 4 correspond to a trapezoid. The choice of these bounds was based on previous research [29], where it was demonstrated that circular and oval cross sections had maintained uniform stress distribution over the stem length while a change from an oval to a trapezoid produced more of a high to low stress distribution which is needed between the neck and prosthesis axes to minimize the stress concentration on the cement at these locations. The lowest and uppermost configurations corresponding, respectively, to the lowest and highest bounds of the search intervals are included in Figure 9. Ten random initial vertices were selected to start the optimization scheme described in Figure 8 and the maximum number of repetitive restarts was set to thirty. The results from each design of experiments along with the associated local optimum were stored for data analysis. Parallel computing with ten processor nodes was used in the FEA and in the calculation of the stress shielding coefficient and critical cement damage accumulation parameter.

### 3.2. Application 1: Titanium Implant

In this application, Titanium alloy (Ti-6Al-4V) with uniform mechanical properties (modulus of elasticity of 110 GPa and a Poisson's ratio of 0.3 [39]) was used for the material of the implant.

**Figure 10:**
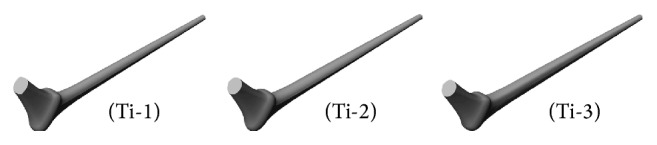
Optimal designs for the Titanium implant.

**Figure 11:**
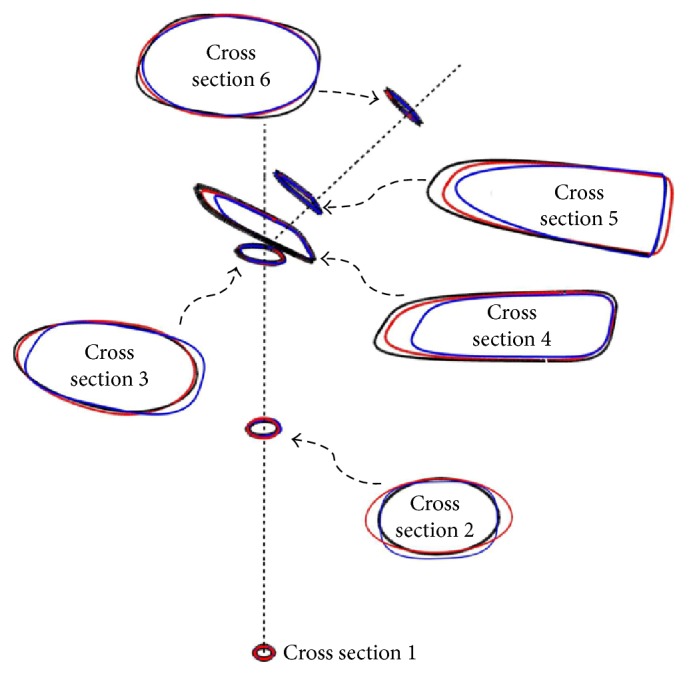
Change in the size and profile of the cross sections for all of Ti-1 (black), Ti-2 (red), and Ti-3 (blue) configurations.

Although several optimal configurations were identified upon the application of the methodology in Section 2, they did not contribute equally to the reduction in stress shielding and critical cement damage accumulation parameter. For brevity we discuss the most common results using three of the optimal configurations as depicted in Figure 10. The exponent (*p*) of the respective cross sections along with the critical cement damage accumulation parameter at 2.0 × 10^6^ cycles is displayed in Table 2. All three designs had comparable critical cement damage accumulation parameters as indicated on the last column which can be attributed to the shape and size of the implant cross sections around the proximal cement. More explicitly, the size of the cross sections had increased and their profile broadened between the implant proximal end and the intersection of the neck and prosthesis axes (sections 6, 5, and 4) as depicted in Figure 11; the wider trapezoidal cross sections in contact with the proximal cement allowed for less stress concentration at these locations eventually delaying damage initiation.

**Figure 12:**
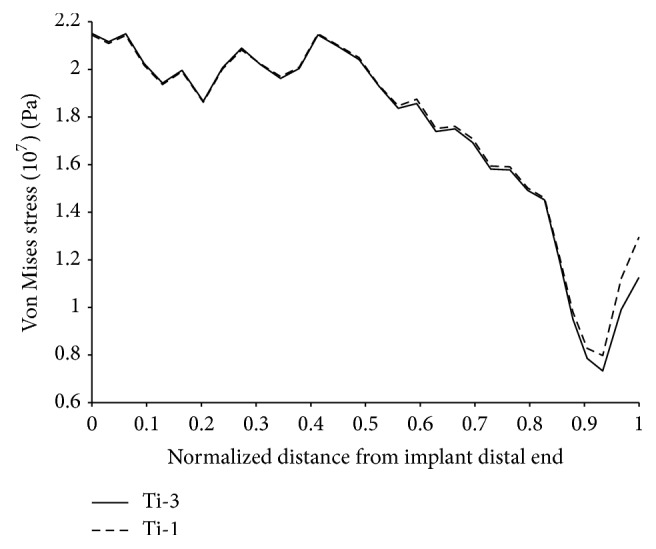
Von Mises stress over the external anterior surface of the bone.

**Figure 13:**
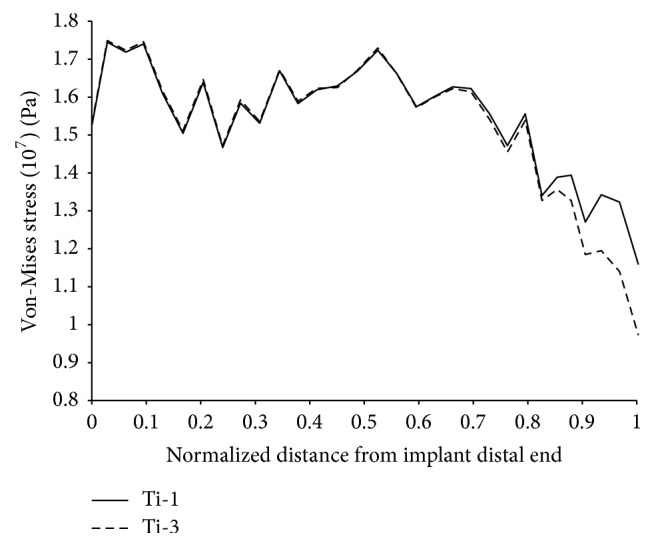
Von Mises stress over the external posterior surface of the bone.

In regard to the stress shielding coefficient, the Ti-1-bone assembly had the smallest value followed by the Ti-2 and Ti-3. The size and profile of the cross sections can also explain these differences as they affect the stiffness of the implant-bone assembly. In the scaled up cross-sectional view in Figure 11, Ti-1 had relatively smaller oval cross sections along the stem length (between cross sections 3 and 1); the relatively slender stem caused the implant-bone assembly to be relatively more flexible to bending. The reduced flexural strength resulted in an increase in the strain energy of the bone upon application of the body and adductor muscle forces which explains the relatively smaller stress shielding coefficient. By contrast, the wider trapezoidal cross sections along the stem length of the Ti-3 configuration caused the bending resistance of the assembly to increase which led to less strain energy in the bone and a relatively larger stress shielding coefficient. To justify these results we plotted the stress distribution (Von Mises stress) over the frontal and posterior external surfaces of the bone in Figures 12 and 13, respectively; there is a small increase in the stress over the proximal region of the bone, more noticeably over the posterior surface as the bending strain is relatively larger in these areas.

Overall, the reduced flexural strength of the implant-bone assembly owing to the slender medial to distal cross sections of the stem was responsible for reducing stress shielding as the strain energy of the bone had increased upon bending; it had also increased the stress over the proximal cement leading to more chances of early damage initiation. Balance was achieved during the global search by using wider trapezoidal cross sections that redistributed the stress and reduced stress concentration at these locations.

### 3.3. Application 2: Functionally Graded Implant

Unlike materials with uniform composition, structural composites, in particular functionally graded materials (FGM), exhibit progressive change in composition, structure, and properties as a function of position within the material. Several processes have been reported to allow fabrication of such composites including plasma spraying, powder metallurgy, and physical vapor deposition [46–48]. A number of studies have demonstrated the suitability of these materials for use in various prostheses including hip, knee, and dental implants [49–52].

In the previous application, we attempted to increase the flexibility of the implant-bone assembly by modifying the profile and size of the stem cross sections. In the current application, a functionally graded material is used for the implant and the optimization technique introduced in Section 2 is applied to further balance stress shielding of the bone and cement damage.

**Figure 14:**
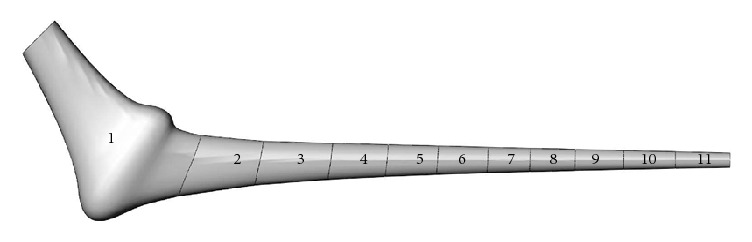
Functionally graded stem with eleven layers.

The functionally graded implant was sliced into eleven lengthwise layers as indicated in Figure 14 and constant material properties were assigned to each layer. Layer #1 in particular had material properties identical to that of Titanium (Ti-6Al-4V) and extended over the proximal cement where the most likely sites of cement damage initiation occur. For the rest of the layers, material stiffness decreased according to the schedule described in Table 3. The length and number of layers were maintained throughout the analysis and Poisson's ratio was assumed constant overall and equal to 0.3. Insight into devising such a model stemmed from previous research [53] where it was demonstrated that stress shielding and interface shear stress were reduced upon decreasing implant material stiffness from the proximal to the distal end of the implant.

**Figure 15:**
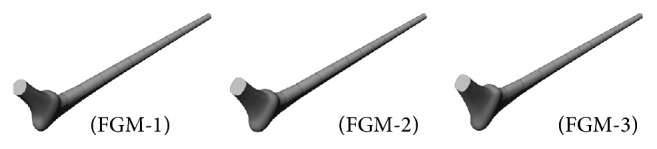
Optimal designs for the functionally graded implant.

**Figure 16:**
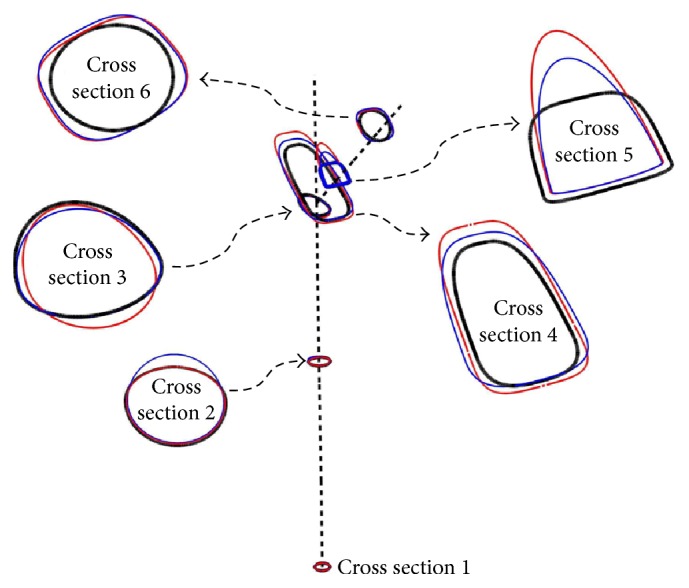
Change in the size and profile of the cross sections for all of FGM-1 (black), FGM-2 (red), and FGM-3 (blue) configurations.

Several optimal configurations were identified upon the application of the methodology in Section 2; in the following we discuss the most common results using three of these designs as depicted in Figure 15. The critical cement damage accumulation parameters along with the exponents (*p*) for all six cross sections are displayed in Table 4. Like the Titanium configurations in Section 3.2, the designs in Figure 15 had oval cross sections over the stem length (cross sections 1 to 3) and trapezoidal cross sections around the intersection of the neck and prosthesis axes; additionally the sizes of the cross sections increased from the proximal end of the implant to the end of the neck axis and decreased afterward toward the distal end of the implant as illustrated in Figure 16.

**Figure 17:**
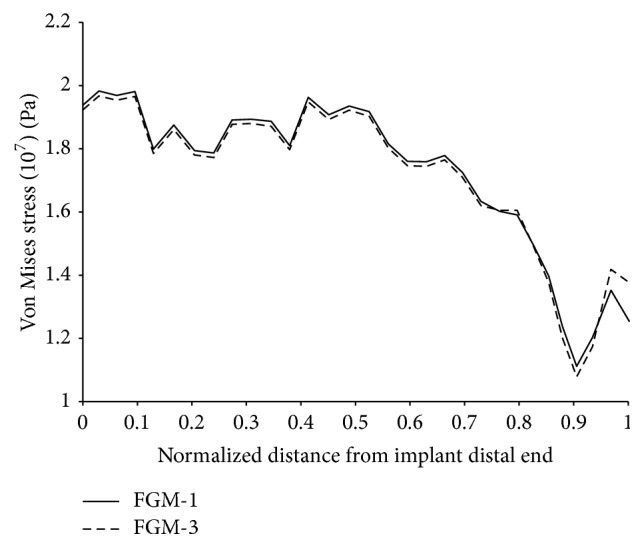
Von Mises stress over the external surface of the bone.

FGM-1 had the smallest stress shielding coefficient followed by FGM-2 and FGM-3 and the size and profile of the cross sections can be used to explain these differences. FGM-1 had relatively smaller cross sections as can be demonstrated from the scaled up view in Figure 16; the smaller cross sections contributed to the implant enhanced flexibility to bending and to that of the assembly all together. As a result, the bone acquired more strain energy upon the application of the body and abductor muscle forces which lead to a relatively smaller stress shielding coefficient. Moreover, the gradual bending of the implant owing to the progressive softening of its material from the proximal to the distal end resulted in less stress over the proximal cement that was further redistributed using the broad trapezoidal cross sections leading to the smaller values in critical cement damage accumulation parameter. The cross sections in FGM-2 and FGM-3 were relatively larger overall resulting in an increase in the bending resistance of the assembly, eventually a relatively larger stress shielding coefficient. To justify these results we plotted in Figure 17 the stress distribution (Von Mises stress) over the external surface of the bone for two of the optimal configurations. There is a small increase in the stress when using FGM-1 implant which explains its relatively smaller stress shielding coefficient. Comparison with the optimal Titanium configurations revealed that FGMs induce less stress shielding and minimum cement damage (Tables 2 and 4).

## 4. Conclusion and Future Work

In this study, we introduced a novel methodology to realistically design cemented hip prostheses by controlling the size and profile of the implant cross sections. The self-regulated technique was used to assess the amount of stress shielding on the bone concurrently with the induced cement damage and then selects a local optimum that equally minimizes their individual effects. In a series of probabilistic restarts a fast global search is implemented and several optimal configurations are found. Stress shielding and cement damage are two conflicting effects nevertheless; a balance can be achieved through geometrical optimization of the implant cross sections. The degree of the balance depends on the material used since gradual softening of the material of the implant induces less stress shielding and minimum damage on the cement. The technique could be improved with a more realistic model of the femur and with accurate and evolutionary models of damage assessment; it can be modified to investigate additional effects such as the change in implant length and surface structure. The methodology can also be extended to other orthopedic joint implants, such as knee and shoulder implants.

## Figures and Tables

**Table 1:** Lower and upper bounds of the exponents over the cross sections in Figure 2.

	Cross section 1	Cross section 2	Cross section 3	Cross section 4	Cross section 5	Cross section 6
Lower bound	2	2	2	2	2	2
Upper bound	2	3	3	4	4	3

**Table 2:** Values of the exponents over the cross sections in Figure 2 along with the critical cement damage accumulation parameter at 2.0 × 10^6^ cycles for three optimal configurations.

Optimal configurations	Exponents						Critical cement damage accumulation parameter (%)
	Cross section 1	Cross section 2	Cross section 3	Cross section 4	Cross section 5	Cross section 6	
Ti-1	2	2	2	4	3	3	2.1
Ti-2	2	2	2	3	3	2	2.9
Ti-3	2	3	3	4	2	2	2.6

**Table 3:** Modulus of elasticity along the length of the functionally graded implant.

Layer #	1	2	3	4	5	6	7	8	9	10	11
Modulus of elasticity (GPa)	120	110	90	80	70	60	50	40	30	20	10

**Table 4:** Values of the exponents over the cross sections in Figure 2 along with the critical cement damage accumulation parameter at 2.0 × 10^6^ cycles for three optimal configurations.

Optimal configurations	Exponents						Critical cement damage accumulation parameter (%)
	Cross section 1	Cross section 2	Cross section 3	Cross section 4	Cross section 5	Cross section 6	
FGM-1	2	2	2	3	4	2	0.4
FGM-2	2	2	2	3	2	3	0.4
FGM-3	2	2	2	4	2	3	0.7

## Appendix

### Variable Variance Probability Density (VVP)

The variable variance probability (VVP) density is based on the minimum distance to the points already sampled and is represented as

(A.1) Φx=12πσ1−edmin2/2σ2dmin=min︸i=1,…,mdi=∑k=1nxk,i−xkxk,u−xk,l2,

where Φ(*x*) is the sampling probability of a point *x*, *n* is the number of design variables, *x*_*i*_ is a point previously sampled, and *m* is the number of points already sampled. Length *d*_*i*_ is the nondimensional distance between point *x* and point *x*_*i*_.

The variance of the normal probability density, which is updated in each restart, is given by

(A.2) $$\begin{array}{c} \sigma =\frac{1}{3\sqrt[{n}]{m}}. \end{array}$$

The variance is gradually decreasing when the number of sampled points is increased.
